# Maternal Isodisomy of Chromosome 3 Combined with a *De Novo* Mutation in the *ABHD5* Gene Causes Autosomal Recessive Chanarin-Dorfman Syndrome

**DOI:** 10.3390/genes12081164

**Published:** 2021-07-29

**Authors:** Julia Kopp, Cristina Has, Alrun Hotz, Sarah C. Grünert, Judith Fischer

**Affiliations:** 1 Institute of Human Genetics, Medical Center, Faculty of Medicine, University of Freiburg, 79106 Freiburg, Germany; Julia.Kopp@uniklinik-freiburg.de (J.K.); Alrun.Hotz@uniklinik-freiburg.de (A.H.); 2 Department of Dermatology, Faculty of Medicine, University Medical Center Freiburg, 79106 Freiburg, Germany; Cristina.Has@uniklinik-freiburg.de; 3 Department of General Pediatrics, Adolescent Medicine and Neonatology, Faculty of Medicine, University Medical Center Freiburg, 79106 Freiburg, Germany; Sarah.Gruenert@uniklinik-freiburg.de

**Keywords:** Chanarin-Dorfman syndrome (CDS), uniparental disomy (UPD), ABHD5, *de novo* mutation

## Abstract

Autosomal recessive Chanarin-Dorfman syndrome (CDS, MIM #275630) is defined as a neutral lipid storage disease with ichthyosis (NLSDI) due to an accumulation of lipid droplets in a variety of different tissues including liver and muscle cells, leucocytes, fibroblasts and nerve cells It is caused by biallelic mutations in the abhydrolase domain containing 5 gene (*ABHD5*, MIM *604780) which is localized on the short arm of chromosome 3. Here we report an 18 month-old girl in whom we have identified the homozygous *ABHD5* mutation c.700C > T, p.(Arg234*). Since none of the parents carried this point mutation, parentage was confirmed by microsatellite marker analysis. Suspected uniparental disomy (UPD) was confirmed by microsatellite genotyping over the entire chromosome 3 and indicated a maternal origin. UPD is an extremely rare event that is not necessarily pathogenic, but may cause disease if the affected chromosome contains genes that are imprinted. Here we report the first case of Chanarin-Dorfman syndrome due to a *de novo ABHD5* mutation in the maternal germ cell, combined with a maternal uniparental isodisomy of chromosome 3. This case demonstrates that genetic analysis of the patient and both parents is crucial to provide correct genetic counseling.

## 1. Introduction

Chanarin-Dorfman syndrome (CDS) is a rare autosomal recessive disorder characterized by ichthyosis. Due to the accumulation of triglycerides in different tissues, other symptoms of the disease can occur: hepatosplenomegaly, mild myopathy, cataract, neurosensory deafness, liver cirrhosis and possible psychomotor development and mental impairment [1]. The presence of lipid droplets in granulocytes, also called Jordan’s anomaly, is the major diagnostic criterion for CDS, and can be found in all patients [1]. Dorfman first described the syndrome in 1974 [2]. The disease is caused by biallelic mutations in the *ABHD5* gene (*abhydrolase domain-containing protein 5, lysophosphatidic acid acyltransferase*, MIM *604780) as first described in 2002 by Lefèvre et al. [3]. To date over 40 different mutations have been published in patients with CDS.

Uniparental disomy (UPD) describes a condition in which an individual carries a pair of chromosomes from the same parent. Several mechanisms are known to lead to (UPD). The presence of two copies of one chromosome in the ovum or sperm cell, which results in a trisomic zygote, is suspected to be the main cause of UPDs. Due to correction of the trisomic state (trisomic rescue), a disomic embryo with two different constitutions can emerge: the embryo either has one chromosome from each parent, or the embryo has two maternal or two paternal chromosomes. A second mechanism for UPD is monosomic rescue, where the monosomic chromosome is replicated. Furthermore, gametic complementation (fertilization of a disomic egg with a nullsomic sperm or vice versa) is possible [4]. In UPD, two constellations are possible: heterodisomy, where both chromosomes of one homologous chromosome pair of one parent are present or isodisomy, where two copies of the same parental chromosome are present.

Here we report a patient with a homozygous mutation in the gene *ABHD5* that emerged from a *de novo* mutation in the maternal germ cells in combination with maternal uniparental isodisomy (UPD) of chromosome 3.

## 2. Materials and Methods

### 2.1. DNA Extraction and Sequencing

Informed consent was obtained and genomic DNA was isolated from peripheral blood lymphocytes of the patient and the parents using standard methods. We performed a next-generation sequencing (NGS) gene panel analysis with a SureSelect XTHS Custom Kit (Agilent Technologies, Inc. Santa Clara, CA, USA) and an Illumina MiSeq system in the patient. All coding exons and flanking intronic sequences of the genes *ABCA12, ALOX12B, ALOXE3, CERS3, CYP4F22, NIPAL4, PNPLA1, SDR9C7, SULT2B1, TGM1* and *ABHD5* were analyzed. Sanger sequencing of the DNA of the patient has validated the *ABHD5* mutation.

### 2.2. Microsatellite Analysis

We performed microsatellite analysis for markers on different chromosomes (D3S1358, THO1, D18S51, PENTA-E, D13S317, D7S820, CSF1PO, PENTA-D, D8S1179, TPOX, FGA) and for 9 markers on chromosome 3 (D3S050, D3S1263, D3S3613, D3S3527, D3S2406, D3S3045, D3S2440, D3S1311, D3S1272) by multiplex PCR and fragment analysis on an ABI Prism 3130XL Automatic DNA Sequencer (Thermo Fisher Scientific, Waltham, MA, USA).

## 3. Results

### 3.1. Clinical Manifestations

**Figure 1:**
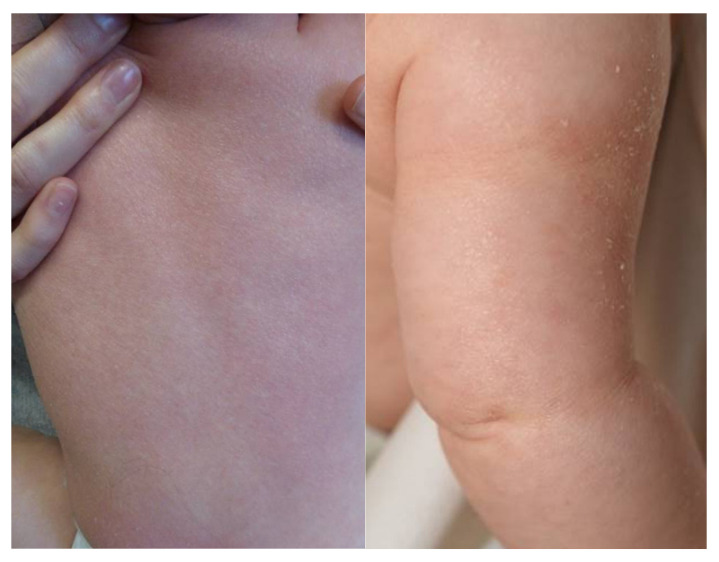
The affected child showed fine whitish scales and mild erythema (on the left: back; on the right: upper arm).

The patient is the first child of non-consanguineous parents with a Caucasian background. Pregnancy was complicated by intrauterine growth retardation in the 3rd trimester, but otherwise unremarkable. The girl was delivered spontaneously in the 40th week of gestation with a birth weight of 2350 g (<1st percentile), birth length was 45 cm (<1st percentile), and head circumference 32.5 cm (<3rd percentile). At birth, her skin was scaly without a collodion membrane. At the age of six months, she presented fine whitish scales and mild erythema (Figure 1).

At the age of 12 months the girl was in good physical condition. Growth was normal with body weight and length at the 17th and 24th percentile, respectively. Apart from mild ichthyosis, slightly delayed psychomotor development and mild axial muscular hypotonia, there was no clinical evidence for other extracutaneous manifestations. The blood count was normal; however, typical lipid vacuoles were present in the leucocytes in a blood smear. Transaminase activities (AST 83 U/L, ALT 45 U/L) and creatine kinase activity (371 U/L) were slightly elevated, whereas triglyceride and total cholesterol levels were normal. Abdominal sonography showed no signs of hepatosplenomegaly or hepatic steatosis. Sonography of the muscles was unremarkable. Echocardiography showed good bilateral function and no signs of cardiomyopathy, and the electrocardiogram was normal. Ophthalmologic examination as well as audiogram and otoacoustic emissions also yielded normal results. Therapeutically, a fat-reduced and fat-modified diet was initiated to replace long-chain fatty acids by medium-chain triglycerides (MCT), and the patient was supported by additional physiotherapeutic measures. Transaminase levels and creatine kinase activity decreased under this regimen, but remained mildly elevated.

### 3.2. Molecular Analysis

Based on the clinical findings, an autosomal recessive congenital ichthyosis (ARCI) was initially suspected. No mutations in the known ARCI genes *ABCA12, ALOX12B, ALOXE3, CERS3, CYP4F22, NIPAL4, PNPLA1, SDR9C7, SULT2B1* and *TGM1* were identified. However, we found the homozygous mutation c.700C > T, p.(Arg234*) in exon 5 of *ABHD5* (NM_016006.6) confirming the molecular genetic diagnosis of CDS. This mutation has previously been described in the literature [5,6] and is clearly defined as pathogenic (class 5) according to the American College of Medical Genetics and Genomics (ACMG) [7]. An overview of all reported pathogenic mutations is listed in Table S1.

**Figure 2:**
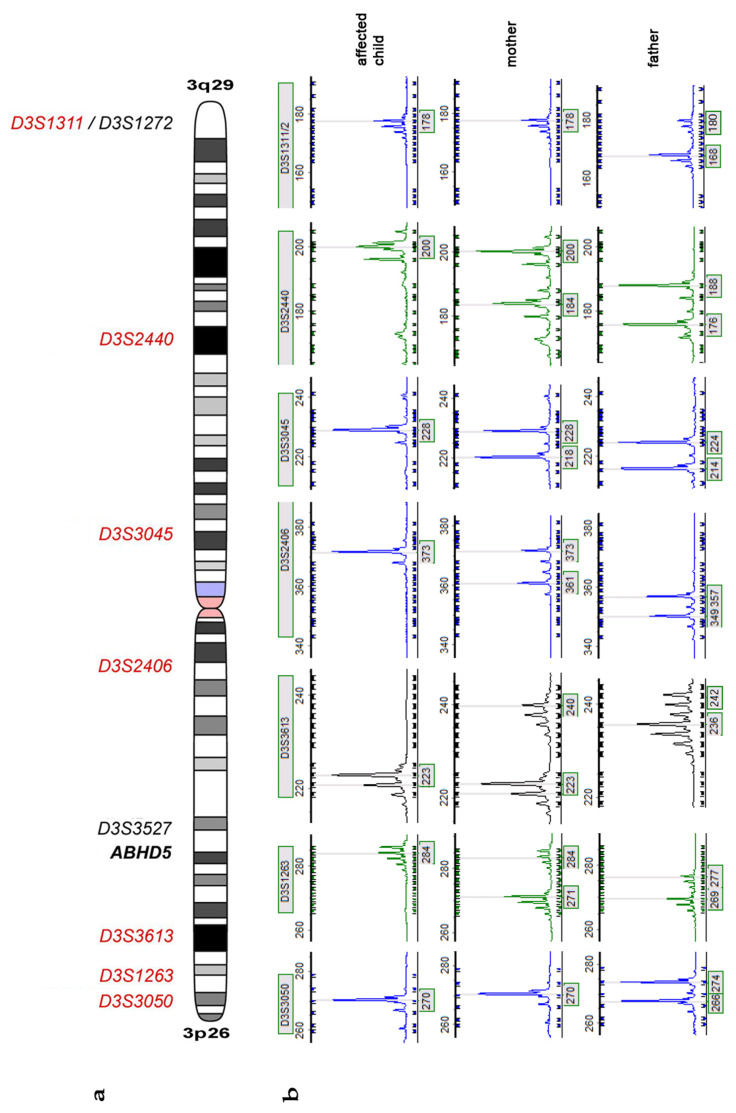
(**a**) Schematic presentation of chromosome 3 and the location of the *ABHD5* gene (bold italic) with the analyzed microsatellite markers (italic); red = maternal UPD in the affected child; black = marker not informative. (**b**) Presentation of Microsatellite marker analysis of all informative markers. The affected child has no paternal allele and therefore did not inherit chromosome 3 of the father. The affected child shows one of the maternal alleles, suggesting a maternal isodisomy.

Since prenatal testing for future pregnancies was requested by the parents, they were screened for the mutation c.700C > T in the *ABHD5* gene. Surprisingly, the mutation was not present in the DNA extracted from the blood of the parents. To exclude mixing up of DNA samples parenthood was confirmed by genotyping a set of microsatellites localized on different chromosomes (Supplementary Materials Table S2). Next we genotyped 9 highly polymorphic microsatellite markers on chromosome 3 (Figure 2a,b and Supp. Table S3) in the patient and her parents. Seven of the 9 markers were informative and clearly showed a maternal origin from the same chromosome in the patient, suggesting a maternal isodisomy of chromosome 3. Furthermore, since the mother did not carry the mutation in the germline, it is likely that it appeared *de novo* in the maternal germ cell.

## 4. Discussion

Here we report a case of Chanarin-Dorfman syndrome due to a *de novo* mutation in *ABHD5* combined with uniparental isodisomy of maternal chromosome 3.

UPD with homozygosity of recessive alleles is being increasingly recognized as the molecular basis for several autosomal recessive disorders, however, data on the clinical prevalence and spectrum of UPD remain limited. Scuffins et al. have recently analyzed 32,067 trio exomes referred for diagnostic testing to create a profile of UPD events and their disease associations [8]. In this cohort, whole-chromosome UPD was observed in 0.31% of cases, resulting in a diagnostic finding in 0.14%. Isodisomy, as found in our patient, was more commonly observed in large chromosomes along with a higher rate of homozygous pathogenic variants [8].

Around 150 genes on specific chromosome portions are known to be imprinted [9], which means, that these genes are expressed differently during development, depending on their maternal or paternal origin. If a UPD occurs in chromosomes that contain imprinted genes, a genetic disease can result from it without the presence of a mutation.

UPD of chromosome 3 has already been reported in the literature, but no imprinting effects have been shown to date. In 2006, Xiao et al. reported a person with a paternal disomy 3 without apparent phenotypic disorders. The authors conclude that there are no paternal imprinted genes on chromosome 3 that cause rare genetic disorders [10].

Nevertheless, UPD of chromosome 3 has been reported in association with diseases. Fassihi et al. described a patient with recessive dystrophic epidermolysis bullosa and a homozygous mutation in the *COL7A1* gene. The mother was heterozygous for the mutation whereas the father did not carry this mutation; microsatellite analysis of chromosome 3 subsequently confirmed a maternal isodisomy of chromosome 3. Since the patient did not show other phenotypic abnormalities, the authors suggested an absence of maternally imprinted genes on chromosome 3 [11]. Hoffman et al. similarly reported a case of Fanconi-Bickel syndrome due to a homozygous *GLUT2* mutation inherited via maternal isodisomy of chromosome 3 [12]. In 2005, Schollen et al. described a patient with a congenital disorder of glycosylation and a homozygous mutation in the *ALG3* gene. Neither the mother nor the father was a carrier of this mutation. Further analysis showed segmental maternal isodisomy of chromosome 3 suggesting that the disease occurred due to a combination of a *de novo* mutation in the *ALG3* gene and segmental isodisomy of chromosome 3 [13].

Our patient showed typical clinical and biological symptoms of a classic CDS such as mild ichthyosis, Jordan abnormalities and mildly elevated transaminases. The child did not present symptoms suggestive of other diseases, which is consistent with the description that there are no maternally imprinted genes on chromosome 3.

Although UPD is a rare cause of autosomal recessive disorders, it has significant implications for both mutation screening and genetic counselling. While the risk of recurrence in subsequent pregnancies is usually 25% for couples with a child affected by CDS, in our case caused by a *de novo* mutation combined with maternal UPD of chromosome 3, the risk is much lower. The precise risk is difficult to estimate; however the recurrence risk of maternal UPD of chromosome 3 is likely less than 1% while the probability for a *de novo ABHD5* mutation is even lower. This emphasizes that genetic testing of the patient and both parents is crucial for correct genetic counselling, not only for dominant disorders more frequently caused by *de novo* mutations but also for autosomal recessive disorders.

## Data Availability

The data presented in this study are available on request from the corresponding author.

## Supplementary Materials

The following are available online at https://www.mdpi.com/article/10.3390/genes12081164/s1, Table S1: mutations in *ABHD5* (NM_016006.6), Table S2: microsatellite analysis for confirmation of parenthood, Table S3: microsatellite analysis for investigation of chromosome 3 UPD.
