# Supplementary material for: Maternal Isodisomy of Chromosome 3 Combined with a De Novo Mutation in the ABHD5 Gene Causes Autosomal Recessive Chanarin-Dorfman Syndrome
Source: Genes (Basel). 2021 Jul 29;12(8):1164. doi: 10.3390/genes12081164 (PMC8391107; doi:10.3390/genes12081164)
Supplement: Supplementary file 1 [file genes-12-01164-s001.zip › genes-1303691-supplementary.pdf]

Table S1: mutations in *ABHD5* (NM_016006.6)

DNA level	Protein level	Reference
c.-3577_47+331delins(26)	p.?	Missaglia *et al.* (2014)
c.19G>A	p.(Glu7Lys)	Lefèvre *et al.* (2001)
c.26del	p.(Asp9Alafs*12)	Cheng *et al.* (2020)
c.46_47del	p.(Arg16Valfs*20)	Lefèvre *et al.* (2001)
c.47+1G>A	p.?	Redaelli *et al.* (2010)
c.98C>G	p.(Ser33*)	Lefèvre *et al.* (2001)
c.134-2A>G	p.?	Lefèvre *et al.* (2001)
c.150C>G	p.(Tyr50*)	Emre *et al.* (2010)
c.217T>G	p.(Ser73Ala)	Emre *et al.* (2010)
c.245A>G	p.(His82Arg)	Schleinitz *et al.* (2005)
c.260G>T	p.(Gly87Val)	Dabas *et al.* (2020)
c.297C>A	p.(Cys99*)	Gupta *et al.* (2016)
c.343A>G	p.(Ser115Gly)	Selma *et al.* (2007)
c.389A>C	p.(Gln130Pro)	Lefèvre *et al.* (2001)
c.413G>A	p.(Trp138*)	Ünlüsoy Aksu *et al.* (2015)
c.506+8113 (insertion LINE-1 element)	p.?	Samuelov *et al.* (2011)
c.507-3C>G	p.?	Srinivasaraghavan *et al.* (2014)
c.507-1G>A	p.?	Bruno *et al.* (2008)
c.550C>T	p.(Arg184*)	Akiyama *et al.* (2003)
c.560_578del	p.(Leu187Glnfs*13)	Nakhaei *et al.* (2018)
c.568C>T	p.(Gln190*)	Lass *et al.* (2006)
c.594dup	p.(Arg199Glnfs*11)	Lefèvre *et al.* (2001)
c.616_646delins(3)	p.(Thr206Glyfs*8)	Pujol *et al.* (2005)
c.662-1330_773+46del	p.?	Redaelli *et al.* (2010)
c.673del	p.(Val225Cysfs*4)	Bruno *et al.* (2008)
c.700C>T	p.(Arg234*)	Schleinitz *et al.* (2005), this study
c.730dup	p.(Thr244Asnfs*10)	Dabas *et al.* (2020)
c.748T>G	p.(Tyr250Asp)	Al-Hage *et al.* (2020)
c.752A>C	p.(His251Pro)	Srinivasan *et al.* (2004)
c.773+1del	p.?	Tamhankar (2014)
c.774-1G>A	p.?	Lefèvre *et al.* (2001)
c.778G>A	p.(Glu260Lys)	Lefèvre *et al.* (2001)
c.811G>A	p.(Gly271Arg)	Eskiocak *et al.* (2019)
c.836del	p.(Gln279Argfs*14)	Shah *et al.* (2017)
c.838C>T	p.(Arg280*)	Takeichi *et al.* (2016)
c.889C>T	p.(Arg297*)	Bruno *et al.* (2008)
c.898_*320del	p.?	Redaelli *et al.* (2010)
c.934C>T	p.(Arg312*)	Pujol *et al.* (2005)
c.960+5G>A	p.?	Redaelli *et al.* (2010)
c.960+6T>A	p.?	Badeloe *et al.* (2008)
c.1006G>T	p.(Glu336*)	Aggarwal *et al.* (2012)

References:

1. Aggarwal S, Maras JS, Alam S, Khanna R, Gupta SK, Ahuja A. Novel nonsense mutation of ABHD5 in Dorfman–Chanarin syndrome with unusual findings: A challenge for genotype–phenotype correlation. *Eur J Med Genet*. **2012**;*55*(3):173-7.

2. Akiyama M, Sawamura D, Shimizu H, Nomura Y, Sugawara M. Truncation of CGI-58 Protein Causes Malformation of Lamellar Granules Resulting in Ichthyosis in Dorfman-Chanarin Syndrome. *J Invest Dermatol*. **2003**;*121*(5):1029-34.

3. Al-Hage J, Abbas O, Nemer G, Kurban M. Chanarin-Dorfman syndrome: a novel homozygous mutation in the ABHD5 gene. *Clin Exp Dermatol*. **2020**;*45*(2):257-9.

4. Badeloe S, van Geel M, Nagtzaam I, Rubio-Gozalbo ME, Oei RL, Steijlen PM, van Steensel MAM. Chanarin–Dorfman syndrome caused by a novel splice site mutation in ABHD5. *Br J Dermatol*. **2008**;*158*(6):1378-80.

5. Bruno C, Bertini E, Di Rocco M, Cassandrini D, Ruffa G, De Toni T, Seri M, Spada M, Li Volti G, D'Amico A, Trucco F, Arca M, Casali C, Angelini C, Dimauro S, Minetti C. Clinical and genetic characterization of Chanarin–Dorfman syndrome. *Biochem Biophys Res Commun*. **2008**;*369*(4):1125-8.

6. Cheng R, Liang J, Li Y, Zhang J, Ni C, Yu H, Kong X, Li M, Yao Z. Next-generation sequencing through multi-gene panel testing for diagnosis of hereditary ichthyosis in Chinese. *Clin Genet*. **2020**; *97*(5): 770-778.

7. Dabas G, Mahajan R, De D, Handa S, Kumar R, Dayal D, Suthar R, Panigrahi I. Managing syndromic congenital ichthyosis at a tertiary care institute-Genotype-phenotype correlations, and novel treatments. *Dermatol Ther*. **2020**;13:e13816.

8. Emre S, Ünver N, Evans SE, Yüzbaşıoğlu A, Gürakan F, Gümrük F, Karaduman A. Molecular analysis of Chanarin-Dorfman syndrome (CDS) patients: Identification of novel mutations in the ABHD5 gene. *Eur J Med Genet*. **2010**;*53*(3):141-4.

9. Eskiocak AH, Missaglia S, Moro L, Durdu M, Tavian D. A novel mutation of ABHD5 gene in a Chanarin Dorfman patient with unusual dermatological findings. *Lipids Health Dis*. **2019**;*18*(1):232.

10. Gupta N, Gothwal S, Satpathy AK, Missaglia S, Tavian D, Das P, Timila D, Kabra M. Chanarin Dorfman syndrome: a case report with novel nonsense mutation. *Gene*. **2016**;*575*(2):359-62.

11. Lass A, Zimmermann R, Haemmerle G, Riederer M, Schoiswohl G, Schweiger M, Kienesberger P, Strauss JG, Gorkiewicz G, Zechner R. Adipose triglyceride lipase-mediated lipolysis of cellular fat stores is activated by CGI-58 and defective in Chanarin-Dorfman Syndrome. *Cell Metab*. **2006**;*3*(5):309-19.

12. Lefèvre C, Jobard F, Caux F, Bouadjar B, Karaduman A, Heilig R, Lakhdar, H, Wollenberg A, Verret JL, Weissenbach J, Ozgüc M, Lathrop M, Prud'homme JF, Fischer J. Mutations in CGI-58, the Gene Encoding a New Protein of the Esterase/Lipase/Thioesterase Subfamily, in Chanarin-Dorfman Syndrome. *Am J Hum Genet*. **2001**;*69*(5):1002-12.

13. Missaglia S, Valadares ER, Moro L, Faguntes EDT, quintão Roque R, Giardina B, Tavian D. Early onset of Chanarin-Dorfman syndrome with severe liver involvement in a patient with a complex rearrangement of ABHD5 promoter. *BMC Med Genet*. **2014**;*15*(1):32.

14. Nakhaei S, Heidary H, Rahimian A, Vafadar M, Rohani F, Bahoosh GR, Amirkashi D. A New Case of Chanarin-Dorfman Syndrome with a Novel Deletion in ABHD5 Gene. *Iran Biomed J*. **2018**;*22*(6):415-419.

15. Pujol RM, Gilaberte M, Toll A, Florensa L, Lloreta J, Gonzalez-Ensenat MA, Fischer J, Azon A. Erythrokeratoderma variabilis-like ichthyosis in Chanarin-Dorfman syndrome. *Br J Dermatol*. **2005**;*153*(4):838-41.

16. Redaelli C, Coleman RA, Moro L, Dacou-Voutetakis C, Elsayed SM, Prati D, Colli A, Mela D, Colombo R, Tavian D. Clinical and genetic characterization of Chanarin-Dorfman Syndrome patients: first report of large deletions in the ABHD5 gene. *Orphanet J Rare Dis*. **2010**;*5*(1):33.

17. Samuelov L, Fuchs-Telem D, Sarig O, Sprecher E. An exceptional mutational event leading to Chanarin-Dorfman syndrome in a large consanguineous family: An exceptional mutational event leading to CDS. *Br J Dermatol*. **2011**;*164*(6):1390-2.

18. Schleinitz N, Fischer J, Sanchez A, Veit V, Harle JR, Pelissier JF. Two new mutations of the ABHD5 gene in a new adult case of Chanarin Dorfman syndrome: an uncommon lipid storage disease. *Arch Dermatol*. **2005**;*141*(6):798-800.

19. Selma ZB, Yilmaz S, Schischmanoff PO, Blom A, Ozogul C, Laroche L, Caux F. A Novel S115G Mutation of CGI-58 in a Turkish Patient with Dorfman–Chanarin Syndrome. *J Invest Dermatol*. **2007**;*127*(9):2273-6.

20. Shah K, Mehmood S, Jan A, Abbe I, Hussain Ali R, Khan A, Chishti MS, Lee K, Ahmad F, Ansar M, Shahzad S, Nickerson DA, Bamshad MJ, Coucke PJ, Santoz-Cortez RLP, Spritz RA, Leal SM, Ahmad W. Sequence variants in nine different genes underlying rare skin disorders in 10 consanguineous families. *Int J Dermatol*. **2017**;*56*(12):1406-13.

21. Srinivasaraghavan R, Krishnamurthy S, Chandar R, Cassandrini D, Mahadevan S, Bruno C, Santorelli FM. Acitretin-Responsive Ichthyosis in Chanarin-Dorfman Syndrome with a Novel Mutation in the ABHD5/CGI-58 Gene. *Pediatr Dermatol*. **2014**;*31*(5):612-4.

22. Srinivasan R, Hadžić N, Fischer J, Knisely AS. Steatohepatitis and unsuspected micronodular cirrhosis in Dorfman-Chanarin syndrome with documented ABHD5 mutation. *J Pediatr*. **2004**;*144*(5):662-5.

23. Takeichi T, Sugiura K, Tso S, Simpson MA, McGrath JA, Akiyama M. Bi-allelic nonsense mutations inABHD5 underlie a mild phenotype of Dorfman-Chanarin syndrome. *J Dermatol Sci*. **2016**;*81*(2):134-6.

24. Tamhankar PM, Iyer S, Sanghavi S, Khopkar U. Chanarin-Dorfman syndrome: clinical report and novel mutation in ABHD5 gene. *J Postgrad Med*. **2014**;*60*(3):332-4.

25. Ünlüsoy-Aksu A, Sarı S, Eğritaş-Gürkan Ö, Dalgıç B. Chanarin-Dorfman syndrome: a novel mutation in a Turkish girl. *Turk J Pediatr*. **2015**;*57*(3):300-3

Table S2: microsatellite analysis for confirmation of parenthood

allele size of the microsatellite markers											
	**TPOX**	**D3S1358**	**FGA**	**CSF1PO**	**D7S820**	**D8S1179**	**THO1**	**D13S317**	**PENTA-E**	**D18S51**	**PENTA-D**
**position**	2:1.493.389-1.493.412	3:45.582.205-45.582.335	4:155.508.848-155.509.043	5:149.455.735-149.456.053	7:83789383-83789484	8:125.907.080-125.907.260	11:2.192.220-2.192.406	13:82.722.059-82.722.243	15:97.374.212-97.374.589	18:60.948.844-60.949.149	21:45.056.398-45.056.421
**affected child**	8/8	17/19	20.2/21	10/10	9/12	15/15	8/11	13/13	9/12	13/16	8/12
**mother**	8/9	17/19	21/22.2	10/12	10/12	15/15	8/8	13/14	11/12	13/17	8/11
**father**	8/8	17/19	20.2/22.2	10/12	9/9	14/15	9.3/11	13/14	9/16	12/16	11/12

Table S3: microsatellite analysis for investigation of chromosome 3 UPD

	**allele size of the microsatellite markers**								
	**D3S3050**	**D3S1263**	**D3S3613**	**D3S3527**	**D3S2406**	**D3S3045**	**D3S2440**	**D3S1272/2**	**D3S1311/2**
**position**	3:3.296.517-3.296.750	3:11.517.252-11.517.482	3:15.361.998-15.362.181	3:39.345.373-39.345.475	3:73.258.381-73.258.698	3:106.989.918-106.990.108	3:147.092.521-147.092.674	3:197.016.169-197.016.398	3:197.018.138-197.018.258
**affected child**	270/270	285/285	222/222	152/152	373/373	228/228	200/200	309/309	178/178
**mother**	270/270	270/285	222/238	152/152	361/373	218/228	184/200	309/309	178/178
**father**	266/274	269/276	234/240	143/152	349/357	214/224	176/188	309/309	168/180
